# Impact of Virtual Reality on Healthcare Provider Empathy for Older Adults with Sensory Impairment

**DOI:** 10.1093/geroni/igab046.2813

**Published:** 2021-12-17

**Authors:** Suzanne Dutton, Andrea Cimino

**Affiliations:** 1 Sibley Memorial Hospital/Johns Hopkins, washington, District of Columbia, United States; 2 Danger Assessment Training and Technical Assistance Center, Johns Hopkins School of Nursing, Baltimore, Maryland, United States

## Abstract

Virtual reality (VR) is an innovative technology that can simulate dual sensory impairment so that healthcare providers can experience this affliction common in older adults. The current study investigated whether VR simulation could increase empathy among healthcare workers. Empathetic care is linked with improved patient satisfaction, compliance, and outcomes. The study used a one-group pre/posttest study design implemented with healthcare providers at a hospital in the Mid-Atlantic region. All participants experienced a 7-minute VR scenario from the viewpoint of “Alfred”, a 74-year-old with macular degeneration and high frequency hearing loss on a commercial VR headset (Oculus Rift). A survey assessed participants’ self-reported knowledge, empathy, and behavior change. Empathy was measured using the validated tool Kiersma-Chen Empathy Scale (KCES). Analyses included descriptive statistics and paired t-tests. Survey results showed that participants increased their knowledge of macular degeneration and hearing loss, and that 9 of 14 empathy items had statistically significant increases (average absolute change = .41 points). Additionally, 97% of participants agreed or strongly agreed that they would utilize the information learned in their work with patients. Evidence suggests VR is an effective intervention to increase empathy and positively change behavior to support persons with sensory impairment.

